# An Extraordinary Instance of the Force of the Hereditary Principle; in Which Is Seen an Example of the Tendency of Every Thing in Nature to Produce Its Like

**Published:** 1839-06

**Authors:** Solyman Brown

**Affiliations:** New-York.


### *An extraordinary instance of the force of the hereditary principle; in which is seen an example of the tendency of every thing in nature to produce its like.*

BY SOLYMAN BROWN, NEW-YORK.

That constitutional peculiarities, whether they be excellencies or defects, are propagated from parent to offspring, even to remote generations is a fact too well established in physical as well as moral science, to admit of doubt. In this respect, the human teeth conform to the general law of nature, as will be evident from the following facts among thousands of a similar character, which are familiar to every experienced Dentist.

In the summer of 1834, the writer of this article, spent a few days in the pleasant village of Frederickton, the capital of the British province of New Brunswick, situated on the River St. John. A lady of that place, presented to me her three little daughters, each of whose left, superior, lateral incisor was black and glossy like polished ebony.

On examining the mouth of the eldest child, who was some twelve years of age, I was so struck with the appearance of a tooth absolutely black, that I put several questions to the mother, the answers to which threw no satisfactory light on the subject. When the second daughter displayed a similar tooth, occupying exactly the corresponding position in the mouth, my surprise was very naturally increased, and absolutely ended in astonishment when the third child showed a precisely similar tooth. Some strange hereditary influence, I was convinced, must have produced this singular phenomenon, and looking anxiously at the mother to ascertain whether she had transmitted the peculiar feature to her offspring, I inquired whether she had such a tooth. As she opened her mouth to answer me, I perceived that her front teeth were white and perfect, and began to doubt the propriety of my surmise, until she put her finger to the place, saying—"In this same spot, I once had a similar tooth, but my parents caused it to be extracted when I was a child."—I found, upon inspection, that her left, upper, small incisor was actually missing, and the adjacent teeth had closed together, leaving the dental arch apparently entire.

Although this example may not, perhaps, be more convincing than a thousand others which are familiar to the members of the profession, it is, at least, unique in its character, and is well fitted to illustrate the well known principle of hereditary predisposition to certain forms of disease.

It is sincerely hoped, that our professional brethren will furnish the Editors of this Journal, from time to time, with statements of anomalous facts which can be well attested, and which illustrate any of the great truths of science.

The remarkable phenomenon described on another page, by Dr. Baker, and which all persons who have seen it, pronounce unparallelled in its singularity, is of this character;—and so incredible is the account of this specimen when committed to paper, but which I can affirm to be wholly true from actual examination, that I am confident, any Dentist or Surgeon will feel himself fully compensated for his trouble, if he will take an opportunity to call on that gentleman, at No. 6 Warren-street, and inspect the tooth personally.

Dentists residing in those sections of the country where it is difficult to obtain drawings of the specimens which they wish to describe in the pages of this Journal, are requested to forward the subject of illustration to either of the Editors by some convenient opportunity, and the necessary drawings will be obtained in this city, Baltimore or Philadelphia, to explain the text.

